# Circulating TGF‐β1 Increases During Vaginal Progesterone Therapy in Women With a Short Cervix

**DOI:** 10.1111/sji.70165

**Published:** 2026-11-05

**Authors:** Zdeněk Laštůvka, Viktor Černý, Anna Škvorová, Petra Petrásková, Olga Novotná, Helena Kopřivová, Jiří Hrdý, Michal Koucký

**Affiliations:** ^1^ Department of Gynecology, Obstetrics and Neonatology General University Hospital in Prague and First Faculty of Medicine, Charles University Prague Czech Republic; ^2^ Institute of Clinical Immunology and Allergology, First Faculty of Medicine Charles University and General University Hospital in Prague Prague Czech Republic; ^3^ Department of Medical Biochemistry and Laboratory Diagnostics, First Faculty of Medicine Charles University General University Hospital in Prague Prague Czech Republic

**Keywords:** preterm labour, progesterone, TGF‐β1

## Abstract

Progesterone is an effective tool for the secondary prevention of spontaneous preterm birth. The aim of this prospective exploratory pilot study was to evaluate the effect of progesterone administration on the number of regulatory T cell (Treg) populations and on the concentrations of the immunoregulatory cytokines IL‐10 and TGF‐β1 during in vivo progesterone treatment in women at risk of preterm delivery. Pregnant women with a singleton pregnancy (22–30 weeks of gestation) who were at risk of spontaneous preterm delivery and required progesterone therapy were included (*n* = 21). Peripheral blood was sampled before progesterone administration and twice thereafter during treatment. Seventeen women attended the second visit, while only seven attended the third visit. The control group consisted of prospectively recruited low‐risk women (*n* = 8). Treg numbers were determined by flow cytometry, and plasma concentrations of TGF‐β1 and IL‐10 were measured by ELISA. In women at risk, plasma TGF‐β1 concentrations increased significantly between the first and second sampling (*p* ≤ 0.01) and remained at a similar level at the third sampling. In contrast, TGF‐β1 levels in the control group remained low without statistically significant change. IL‐10 concentrations were generally low in both groups. This pilot study was associated with an increase in the immunoregulatory cytokine TGF‐β1 following progesterone administration. Although TGF‐β1 is produced by several cell types in addition to Tregs, our findings raise the possibility that progesterone reinforces an immunoregulatory pathway relevant to the maintenance of pregnancy in women at risk of preterm birth; however, the present study does not establish this mechanism.

## Graphical abstract

Progesterone treatment was associated with increased plasma TGF‐β1 concentrations in pregnant women at risk of spontaneous preterm birth. These findings suggest that progesterone may enhance immunoregulatory pathways relevant to pregnancy maintenance, although the underlying mechanism remains to be established.

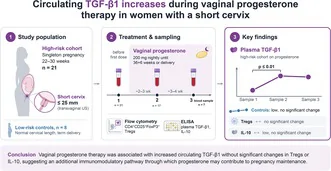

**Abbreviations**
ANOVA: analysis of variance
APC: allophycocyanin
CD: cluster of differentiation
EDTA: ethylenediaminetetraacetic acid
ELISA: enzyme‐linked immunosorbent assay
FITC: fluorescein isothiocyanate
FoxP3: forkhead box P3
IL: interleukin
IL‐10: interleukin‐10
iTreg: induced regulatory T cell
LAP: latency‐associated peptide
LTBP: latent transforming growth factor beta‐binding protein
nTreg: natural regulatory T cell
PE: phycoerythrin
PIBF: progesterone‐induced blocking factor
pPROM: preterm prelabor rupture of membranes
PR: progesterone receptor
Smad: a family of structurally similar proteins that are the main signal transducers for receptors of the transforming growth factor beta
TGF‐β: transforming growth factor beta
TGF‐β1: transforming growth factor beta 1
Treg: regulatory T cell

## 1 Introduction

During pregnancy, the maternal organism must support the development of a semiallogeneic fetus and simultaneously tolerate foreign paternal antigens while maintaining defence against pathogens. This state is achieved through a dynamic balance of complex immuno‐endocrine interactions [1].

The successful course of pregnancy requires, among other factors, the coordinated function of multiple components of the immune system at both the local level (in the decidua) and the systemic level. Various immune cell populations with specialized characteristics contribute to establishing and maintaining the tolerogenic state of the maternal organism during pregnancy. In studies addressing the immunological background of preterm birth, these immune populations are analysed in terms of their number, phenotype, and cytokine production profiles. Of particular importance is the population of regulatory T lymphocytes (Tregs) [2, 3].

At present, the role of regulatory T lymphocytes in normal human pregnancy can be evaluated only through analysis of maternal peripheral blood. The number of Tregs physiologically increases during gestation, and their decline has been associated with both term and preterm labor [4].

An adequate number and proper function of Tregs play a crucial role in preventing several pregnancy complications, including spontaneous abortion, spontaneous preterm labor, and preeclampsia [5, 6, 7].

Tregs regulate the immune system through a variety of local and systemic mechanisms, including the induction of apoptosis in autoreactive cells and the production of tolerogenic cytokines, particularly IL‐35, TGF‐β, and IL‐10 [8].

TGF‐β is a multifunctional cytokine [9] with important immunoregulatory functions [10]. It is produced not only by immunocompetent cells but also by various other cell populations, primarily in a latent form in which it forms a complex with two additional polypeptides—latent TGF‐β binding protein (LTBP) and latency‐associated peptide (LAP). Serum proteases such as plasmin catalyse the release of active TGF‐β from this complex.

The key functions of TGF‐β include the regulation of inflammatory processes, particularly at the maternal–fetal interface. In addition, TGF‐β plays a crucial role in stem cell differentiation as well as in the regulation of T cells. It contributes to immune tolerance by inhibiting T‐cell proliferation, promoting the expansion of Tregs, influencing NK‐cell differentiation, and maintaining cellular homeostasis [9]. For these reasons, TGF‐β has been extensively studied in cancer, autoimmune and infectious diseases, and mucosal immunology [10, 11, 12].

TGF‐β exerts its effects primarily through a relatively simple signal transduction pathway mediated by Smad proteins. These proteins transmit the TGF‐β signal from membrane‐bound receptors to the nucleus, where they regulate the transcription of target genes [13].

TGF‐β plays a crucial role in pregnancy. In recent years, research has increasingly focused on the association between TGF‐β levels and pregnancy complications. TGF‐β signalling is essential for the differentiation of extravillous trophoblast cells and is predominantly localized in the cytoplasm of villous syncytiotrophoblasts and extravillous trophoblasts throughout gestation. It contributes to successful embryo implantation and limits excessive invasion of extraepithelial trophoblasts. Elevated TGF‐β levels have been associated with preeclampsia and gestational diabetes, whereas decreased levels may predispose to recurrent miscarriage due to a compromised environment of immune tolerance [14, 15, 16, 17].

Beyond its role in maternal–fetal tolerance and placental development, TGF‐β is also relevant to parturition itself. Labor at term and preterm shares common features, namely increased uterine contractility, cervical dilatation, and rupture of the fetal membranes, and involves the fetal membranes, decidua, and myometrium, with inflammation being a key mechanism [18, 19]. The effects of TGF‐β depend on the cellular context [9], and TGF‐β signalling has been implicated in several of these tissues. In the human myometrium, TGF‐β receptor types I and II are upregulated in pregnant non‐laboring tissue compared with non‐pregnant myometrium and decrease in spontaneously laboring tissue [20], and expression of the Type II receptor is reduced in myometrium from women in preterm labor complicated by chorioamnionitis [21]. Phosphorylated (active) Smad3 is decreased in the myometrium after spontaneous term labor, and Smad3 knockdown in primary human myometrial cells augments the inflammation‐induced production of cytokines, chemokines, and prostaglandin F_2α_, indicating that Smad3 restrains pro‐inflammatory and pro‐labor mediators in vitro [22]. In addition, the inhibitory Smad7 is increased in myometrium and fetal membranes after spontaneous term labor, and its silencing reduces inflammation‐induced pro‐labor mediators [23]. In human fetal membranes, TGF‐β reduced basal TNF‐α release from cultured amniochorion, although it had little effect on lipopolysaccharide‐stimulated TNF‐α production [24], and in a rabbit model, intra‐amniotic TGF‐β2 prevented preterm delivery induced by interleukin‐1α and TNF‐α [25]. In the rat cervix, TGF‐β1 expression and Smad2 activation increase during gestation and decline shortly before parturition, coinciding with the transition to cervical ripening [26]. Taken together, these data suggest that the role of TGF‐β in pregnancy is not limited to immune tolerance and that TGF‐β signalling may be involved at several levels of the parturition cascade.

To date, no data are available on the longitudinal assessment of circulating TGF‐β1 during vaginal progesterone administration in women with a short cervix and relationship with spontaneous preterm birth. Spontaneous preterm labor remains a major socioeconomic and medical challenge, affecting approximately 10% of births worldwide. In women at increased risk of preterm birth, progesterone administration has been successfully implemented in clinical practice. A history of spontaneous preterm labor and/or the presence of a short cervix is the main indications for obstetricians to initiate progesterone therapy. Consequently, additive progesterone administration in women at risk of preterm birth is currently regarded as an effective measure for secondary prevention of this serious pregnancy complication [27, 28, 29, 30].

Progesterone is well known for its numerous endocrine functions during pregnancy, but it also possesses important immunomodulatory properties. It participates in the regulation of inflammatory responses, activation of immune cells, and production of cytokines—key mediators of immune adaptation to pregnancy. A growing body of evidence supports the crucial role of endogenous progesterone in the maintenance of normal gestation. Many of its immunological effects are mediated through a downstream effector known as the progesterone‐induced blocking factor (PIBF) [31]. For example, progesterone has been shown to exert a tolerogenic immune effect by enhancing the phagocytic capacity of trophoblasts and increasing the expression of anti‐inflammatory mediators, such as TGF‐β, produced by trophoblast‐derived cells [32]. However, little is known about how additive progesterone administration influences pregnancy well‐being in women at risk of preterm birth.

Given these immunomodulatory properties, we reasoned that vaginal progesterone might reinforce immunoregulatory pathways capable of modulating components of the inflammatory program of parturition. The aim of this study was to evaluate the effect of progesterone therapy on the number of regulatory T cell (Treg) populations and the concentrations of the immunoregulatory cytokines IL‐10 and TGF‐β1 during in vivo progesterone treatment in women at risk of preterm delivery.

## 2 Materials and Methods

### 2.1 Patients

This was a prospective study involving a cohort of high‐risk patients enrolled at the Department of Gynaecology, Obstetrics and Neonatology, General University Hospital in Prague, and the First Faculty of Medicine, Charles University, between January 2024 and February 2025. Pregnant women with a singleton pregnancy recruited from the Maternal–Fetal Unit met the inclusion criterion of a short cervix, defined as a cervical length ≤ 25 mm. Cervical length was assessed by transvaginal ultrasound using standard equipment (Acuson XP128, Mountain View, CA, USA). All examinations were performed by an experienced and certified sonographer (Fetal Medicine Centre, London, UK). Transvaginal cervical measurements were obtained following the technique described by Iams et al. [33].

After blood sample collection, patients with a short cervix were prescribed vaginal progesterone (200 mg nightly) until the completion of the 36th week of gestation or until delivery, whichever occurred first. Gestational age was determined based on the date of the last menstrual period and confirmed by a first‐trimester ultrasound scan. The high‐risk cohort comprised 21 women recruited between 22 and 30 weeks of gestation. All women with a short cervix were asymptomatic at inclusion, with no regular uterine contractions and no cervical dilatation; women with ruptured membranes or an ongoing infection were not eligible, as specified in the exclusion criteria. Ten of the 21 women (47.6%) received antenatal corticosteroids and tocolysis later in pregnancy, after all study samples had been obtained; in these women, the last study sample preceded delivery by a median of 14 days (range 8–49 days). The control group included 8 pregnant women with uncomplicated pregnancies and normal cervical length who attended routine prenatal care at the same department. Control women were recruited prospectively, before their pregnancy outcome was known, and no woman was excluded from the control group on the basis of pregnancy outcome; all eight control women delivered at term.

The following exclusion criteria were applied to all participants:

– with multiple pregnancies,
– suffering from any autoimmune disease, including Type I diabetes,
– with a history of/ongoing cancer‐related illness,
– with untreated or decompensated allergies, asthma,
– ongoing infectious disease,
– with amniotic fluid leakage (pPROM; preterm prelabor rupture of membranes).

Three repeated collections from the cubital vein were performed at the following time intervals:

– 1st collection: at the time before the first vaginal administration of progesterone (*n* = 21),
– 2nd collection: after approximately 2–3 weeks of progesterone administration (*n* = 17),
– 3rd collection: approximately after a further 3–4 weeks of progesterone administration (*n* = 7).

Pregnant women in the control group also had blood samples drawn from the cubital vein at three time points during pregnancy:

– 1st collection at 25th–26th week of pregnancy (*n* = 8),
– 2nd collection in the ongoing 30th–31st week of pregnancy (*n* = 6),
– 3rd collection during the 35th–36th week of pregnancy (*n* = 5).

These time intervals were determined to correspond most closely to the weeks of pregnancy in which high risk women were most frequently sampled. The gestational age at each collection is given in Table 1. In the high‐risk cohort, the median interval between the first and the second collection was 18 days (range 14–22) and between the second and the third collection was 25 days (range 20–28).

**TABLE 1:** Patient details.

	High risk women (*n* = 21)	Controls (*n* = 8)
Age, years	31 (27–35)	30 (26–34)
BMI, kg/m^2^	26 (20–32)	23 (19–25)
Smoking, *n* (%)	6 (28.6)	0 (0)
IVF conception, *n* (%)	5 (23.8)	0 (0)
Cervical length at inclusion, mm	19 (12–24)	34.5 (29–42)
Gestational age at 1st collection	27 + 0 (22 + 0–30 + 0)	25 + 3 (25 + 1–25 + 5)
Gestational age at 2nd collection	30 + 1 (26 + 6–32 + 0)	30 + 2 (30 + 1–30 + 5)
Gestational age at 3rd collection	34 + 2 (30 + 2–35 + 1)	35 + 3 (35 + 1–35 + 5)
Gestational age at delivery	35 + 0 (26 + 0–41 + 3)	39 + 2 (37 + 5–40 + 5)
Preterm birth < 37 + 0 weeks, *n* (%)	13 (61.9)	0 (0)
Obstetric history, *n* (%)		
Previous spontaneous preterm birth	13 (61.9)	0 (0)
Previous second‐trimester loss	1 (4.8)	0 (0)
Previous term delivery only	3 (14.3)	5 (62.5)
No previous delivery	4 (19.0)	3 (37.5)
Antenatal corticosteroids and tocolysis, *n* (%)	10 (47.6)	0 (0)

*Note:* Descriptive characteristics of the high risk cohort (*n* = 21) and the controls (*n* = 8). Values are expressed as median (range) or *n* (%) unless otherwise indicated. Gestational age is given as completed weeks + days. Blood was collected from 21, 17 and 7 high risk women and from 8, 6 and 5 controls at the first, second and third collection, respectively; gestational ages at each collection refer to the women actually sampled. All high risk women had a cervical length ≤ 25 mm and were asymptomatic at inclusion. Antenatal corticosteroids and tocolysis were administered later in pregnancy, after all study samples had been obtained. Obstetric history categories are mutually exclusive. In the controls, the median gestational age at delivery fell between 39 + 1 and 39 + 2 weeks and is reported as 39 + 2. No formal statistical comparisons between the groups were performed.

### 2.2 Ethical Considerations

This research was approved by the Ethics Committee of the First Faculty of Medicine, Charles University, Prague, Czech Republic (No 481/19, S‐IV). The study was conducted in accordance with the principles of the Declaration of Helsinki. Signed informed consent was obtained from all participants.

### 2.3 Blood Sampling

Peripheral blood (6 mL) was collected into EDTA tubes (Vacutainer, Becton Dickinson, San José, CA, USA) and analysed by flow cytometry within 5 h of collection. Aliquotes for TGF‐β1 detection were obtained from EDTA tubes within 30 min from blood collection using two‐step centrifugation (first centrifugation speed 3000 × *g* for 10 min, 4°C and the second centrifugation at speed 12,000 × *g* for 10 min at 4°C). Aliqutes were stored at −80°C until further processed. All personal data of the participants were anonymized.

### 2.4 Flow Cytometry Analysis/Gating Strategy

The absolute Treg cell count was calculated from the absolute number of CD4^+^CD25^+^ T lymphocytes, which was determined using a BD FACSCanto flow cytometer and BD FACSCanto clinical software with BD Trucount Absolute Counting Tubes (cat. no. 340334; BD Biosciences, USA), according to the following protocol. Briefly, 50 μL of freshly mixed whole blood was added to a BD Trucount tube containing antibodies against CD25 (PE, clone MEM‐241) and CD4 (FITC, clone MEM‐181), together with 5 μL of anti‐CD45 antibody (PerCP, clone 2D1). An additional 5 μL of PBS was added to adjust the total volume. The sample was vortexed to ensure thorough mixing with the Trucount bead pellet and incubated for 15 min at room temperature in the dark. Subsequently, 450 μL of BD FACS Lysing Solution was added, and the sample was immediately analysed without washing using a “lyse‐no‐wash” protocol.

The proportion of Treg was determined using a combination of cell surface markers (CD4, CD25) and the transcription factor FoxP3, as previously described [34, 35, 36]. Briefly, 100 μL of peripheral blood was stained with antibodies against CD4 conjugated to FITC (clone MEM‐241) and CD25 conjugated to PE (clone MEM‐181). Cells were then fixed and permeabilized using the Human Regulatory T Cell Whole Blood Staining Kit (eBioscience, cat. no. 88‐8996) according to the manufacturer's instructions. Subsequently, anti‐FoxP3 antibody (clone PCH101) conjugated with APC was added. After a 30‐min incubation, samples were washed three times and immediately acquired on a BD FACSCanto II flow cytometer (BD Biosciences, San Jose, CA, USA). Flow cytometry data were analysed using FlowJo software, followed by statistical and graphical evaluation in GraphPad Prism. The gating strategy and representative dot plots are shown in Figure 1.

**FIGURE 1:**
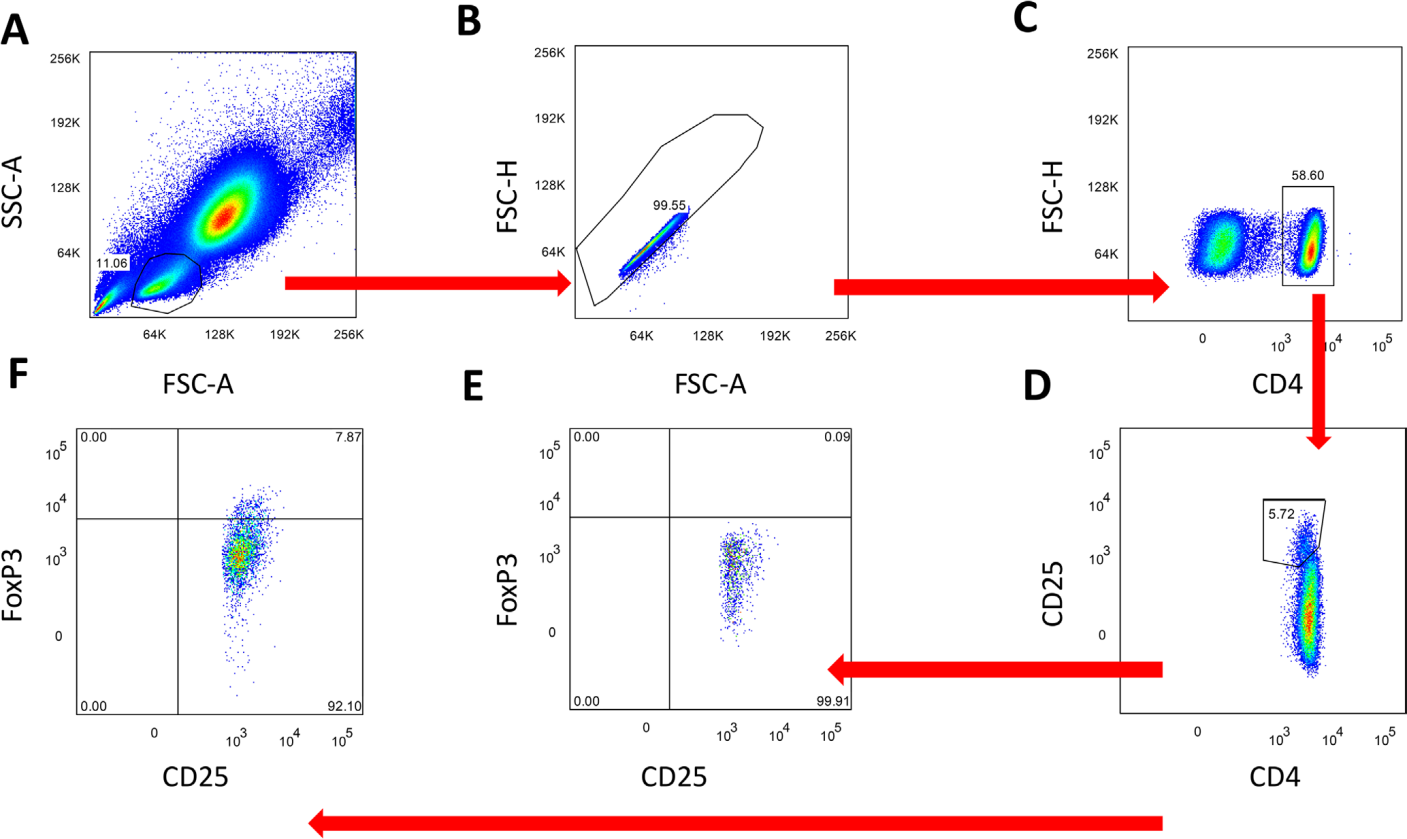
Gating strategy for Treg determination. Population of lymphocytes was selected (A) followed by doublets exclusion (B). CD4^+^ T cells were identified (C) and then CD4^+^CD25^+^ cells were selected (D). Fluorescence minus one control (E) was used to set a gate for CD4^+^CD25^+^FoxP3^+^ cells as shown in (F).

Plasma was collected, aliquoted, and stored at −80°C until cytokine measurement (only one freeze–thaw cycle of samples to determine cytokine concentration was included). The concentrations of key immunoregulatory cytokines (TGF‐β1 and IL‐10) were determined by ELISA as previously described [36]. Briefly, IL‐10 levels were quantified using an ELISA kit (R&D Systems, DY217B), and TGF‐β1 was measured using a primary monoclonal antibody (MAB240), a biotinylated secondary antibody (BAF240), and recombinant protein (240‐B) to generate a standard curve. Standard calibration curve was calculated and data were analysed using software KIM (Schoeller Instruments, Prague, Czech Republic). All samples were assayed in duplicates, and detection limits were as follows: 31.25 pg/mL for IL‐10 and 15.625 pg/mL for TGF‐β1. Intraassay coefficient of variability (CV) was 6.57%, and interassay CV was 10.38%. To activate latent TGF‐β1 to an immunoreactive mature form, all plasma samples were acidified using 2.5 acetic acid and 10 M urea for 10 min followed by neutralization with 2.7 N NaOH and 1 M HEPES for an additional 10 min.

### 2.5 Statistics

All data were statistically and graphically analysed using GraphPad Prism software, version 6.01 (GraphPad Software, San Diego, CA, USA). One‐way ANOVA followed by Tukey's post hoc test was applied to evaluate differences in Treg population proportions and absolute cell counts obtained from flow cytometry. The Kruskal–Wallis test followed by Dunn's multiple comparison test was used for statistical analysis of cytokine levels measured by ELISA. As an exploratory analysis, paired TGF‐β1 concentrations between the first and second sample were compared using the Wilcoxon signed‐rank test.

## 3 Results

During the follow‐up of the high‐risk cohort (*n* = 21), 13 women delivered prematurely, i.e., before 37 completed weeks of gestation. All preterm births occurred spontaneously between 26^+0^ and 36^+6^ weeks of gestation. Throughout the study period, no fetal deaths or other major pregnancy complications were observed.

The median maternal age at enrollment was 31 years (range, 27–35 years) in the high‐risk cohort and 30 years (range, 26–34 years) in the control cohort. Maternal age was similar in the two groups (Table 1).

Descriptive characteristics of the high‐risk cohort (*n* = 21) and control group (*n* = 8) are presented in Table 1. Values are expressed as median (range) or *n* (%) unless otherwise indicated. No formal statistical comparisons between the groups were performed.

The median gestational age at the first, second, and third collection was 27 + 0, 30 + 1, and 34 + 2 weeks in the high‐risk cohort and 25 + 3, 30 + 2, and 35 + 3 weeks in the control group (Table 1). The median cervical length at inclusion was 19 mm (range 12–24 mm); 14 women (66.7%) had a cervical length ≤ 20 mm and 7 women (33.3%) 21–25 mm. A previous spontaneous preterm birth was documented in 13 of the 21 women (61.9%); one woman had a previous second‐trimester loss at 21 weeks, three women had only previous term deliveries, and four women had a history of recurrent early pregnancy loss without a previous delivery.

### 3.1 Detection of Tregs in Peripheral Blood of Women at Risk of Preterm Labor by Flow Cytometry

No statistically significant difference was found in either the percentage or absolute number of CD4^+^CD25^+^FoxP3^+^ Tregs in the peripheral blood of women at increased risk of preterm birth during progesterone therapy (Figures 2 and 3).

**FIGURE 2:**
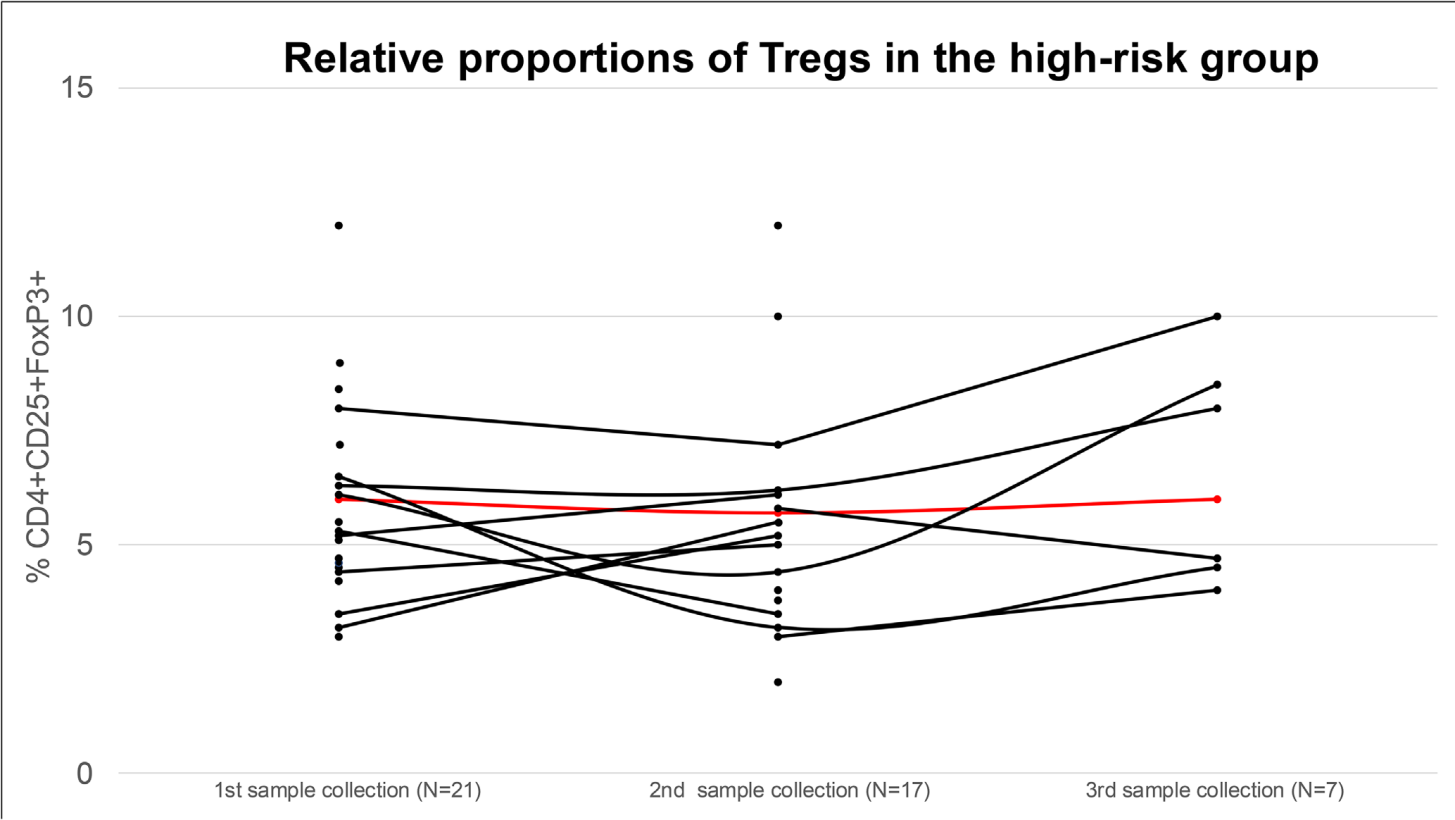
Comparison of relative proportions of Tregs in the high‐risk group at the three sample collection timepoints. The results were statistically evaluated by one‐factor ANOVA followed by Tukey's post hoc test. Treg, regulatory T lymphocytes; *n*, number of women.

**FIGURE 3:**
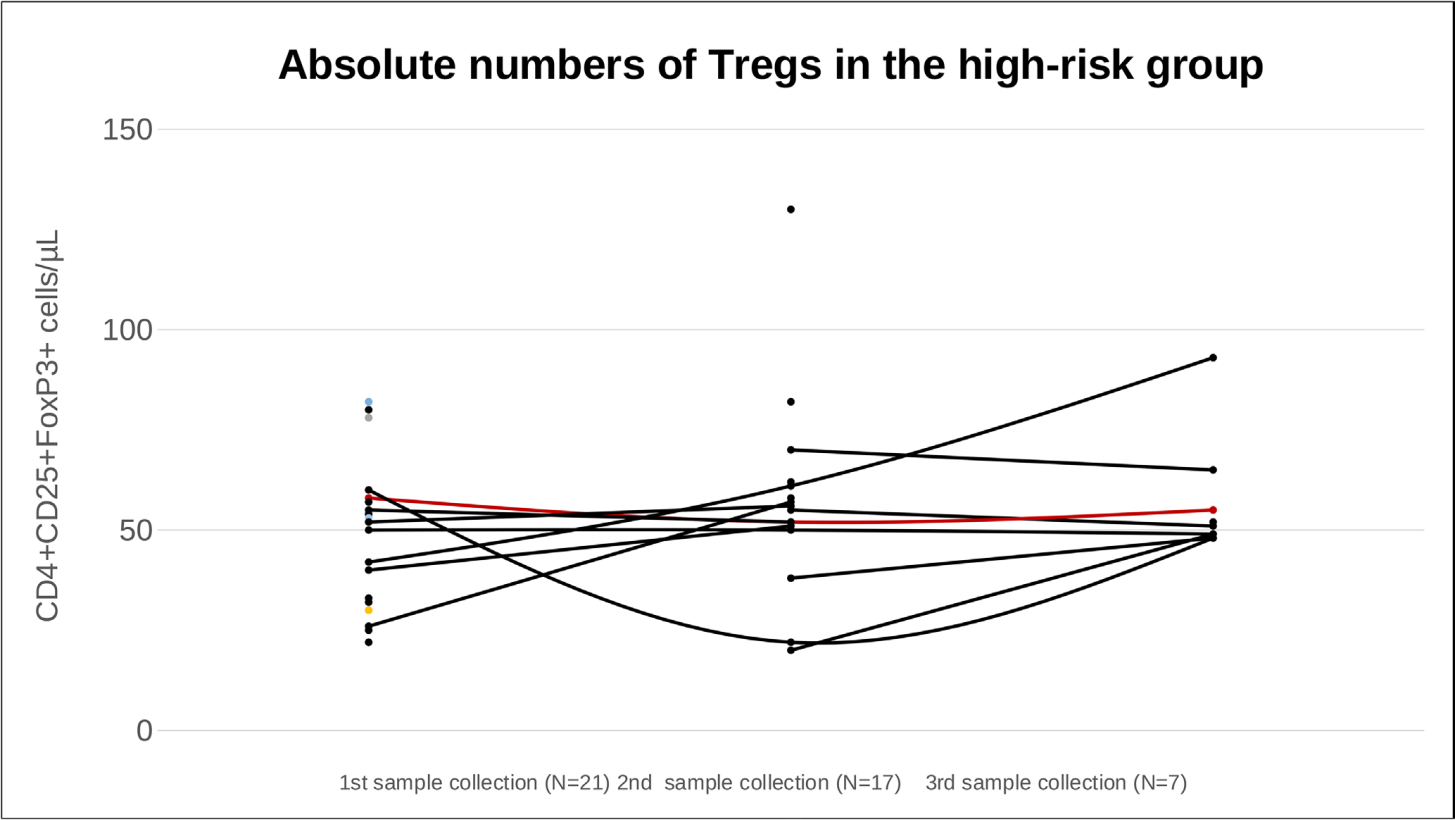
Comparison of absolute numbers of Tregs in the high‐risk group at the three sample collection timepoints. The results were statistically evaluated by one‐factor ANOVA followed by Tukey's post hoc test. Treg, regulatory T lymphocytes; *n*, number of women.

### 3.2 Determination of IL‐10 and TGF‐β1 in Blood Plasma of High‐Risk and Low‐Risk Women by ELISA

Plasma concentrations of IL‐10 and TGF‐β1 were measured in women at increased risk of preterm birth during progesterone therapy. Corresponding concentrations of IL‐10 and TGF‐β1 were also determined in women at low risk of preterm birth during pregnancy.

In high‐risk women, plasma TGF‐β1 concentrations increased significantly between the first and second sampling (*p* ≤ 0.01) and remained at a similar level at the third sampling (Figure 4A). In the control group of low‐risk women, TGF‐β1 concentrations (Figure 4B) were low and showed a non‐significant increasing trend throughout pregnancy.

**FIGURE 4:**
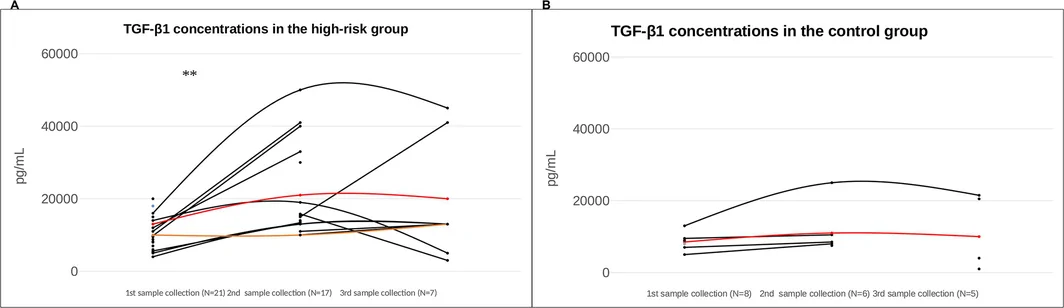
Concentrations of TGF‐β1 in the high‐risk group (A) and in the control group (B) at the three sample collection timepoints, shown on the same scale. The Kruskal–Wallis test followed by Dunn's multiple comparison test was used for statistical evaluation of the data. ELISA, enzyme‐linked immunosorbent assay; *n*, number of samples; TGF‐β1, transforming growth factor β1, **p ≤ 0.01.

IL‐10 concentrations were generally low and showed no statistically significant differences between sampling points in either the high‐risk or control groups. As an exploratory analysis, paired TGF‐β1 concentrations between the first and second sample were compared using the Wilcoxon signed‐rank test. The analysis suggested an overall increase in TGF‐β1 concentrations between the two sampling time points; however, given the small sample size and the exploratory nature of the analysis, this finding should be interpreted cautiously and should be a part of follow‐up confirmatory study on a substantially larger group of patients.

## 4 Discussion

In this exploratory study in women with a short cervix, circulating TGF‐β1 increased significantly between the first and the second sampling during vaginal progesterone treatment and remained elevated at the third, whereas neither the proportion nor the absolute number of circulating CD4^+^CD25^+^FoxP3^+^ Tregs changed. This dissociation is the central observation of the present study, and the Discussion is organized around it. Our design does not permit any conclusion about the efficacy of progesterone in preventing preterm birth: all women with a short cervix received progesterone, no untreated comparison group was available, and 13 of the 21 women (61.9%) nevertheless delivered before 37 weeks of gestation.

Spontaneous preterm birth, together with preeclampsia, represents one of the major obstetric syndromes, affecting nearly 10% of pregnant women worldwide. Primary prevention is not yet available. Among the most effective measures for secondary prevention is vaginally administered micronized progesterone. This form of progesterone is recommended for women at risk of preterm birth, particularly those with a history of spontaneous preterm delivery or a short cervix [37, 38, 39].

In both situations, vaginal progesterone has been shown to significantly reduce the risk of spontaneous preterm birth [40, 41]. Numerous studies have examined the actions of endogenous progesterone in human tissues. Several theories have been proposed, including one put forward by Menon [19]. The progesterone receptor (PR) is expressed in a wide range of human tissues, both reproductive and non‐reproductive. Spontaneous preterm birth is thought to be preceded by complex alterations in progesterone receptor expression and function within the myometrium [19].

Progesterone has well‐characterized endocrine functions during pregnancy but also exerts important immunomodulatory effects. It influences the induction of inflammatory responses, the activation of immune cells, and the production of various cytokines [31]. Despite many years of research, the mechanism by which additive vaginal progesterone reduces the risk of preterm birth remains unclear. Various studies have suggested that progesterone may influence both immune and non‐immune cell populations, as well as the composition of the microbiota. However, the exact mechanism—or combination of mechanisms—remains controversial [42].

Our group has previously demonstrated that progesterone administration reduces colonization of the birth canal by *Ureaplasma urealyticum* [43]. The findings of our earlier work are consistent with those of a recent experimental study in a mouse model, which demonstrated a relationship between progesterone administration and dysbiotic changes in the vaginal microbiota. Progesterone treatment led to decreased production of TNF‐α and IL‐6, indicating a reduction in the inflammatory response. The authors suggested that analysis of microbial colonization before conception or in early pregnancy may aid in clinical decision‐making regarding progesterone administration [44].

In the present study, we investigated the relationship between additive vaginal progesterone administration and the number of regulatory T lymphocytes, as well as the concentrations of immunoregulatory cytokines, in women at risk of preterm delivery. In contrast to previous reports, we did not observe any significant changes in Treg counts within our cohort [45]. An interesting finding was the increase in circulating TGF‐β1 in the absence of a detectable increase in the proportion of circulating CD4^+^CD25^+^FoxP3^+^ Treg cells. This dissociation suggests that the observed increase in TGF‐β1 cannot be explained simply by expansion of the circulating Treg compartment. TGF‐β1 is produced by a broad range of immune and non‐immune cell types, including other lymphocyte and myeloid populations, platelets, endothelial and stromal cells, as well as pregnancy‐associated tissues. Therefore, several potential cellular or tissue sources could contribute to the circulating TGF‐β1 signal observed in our study. However, because the cellular origin of TGF‐β1 was not directly investigated, our data do not allow attribution of the observed increase to any specific source.

Several considerations may explain why circulating TGF‐β1 increased without a parallel change in circulating Tregs. First, plasma TGF‐β1 cannot be attributed to Tregs: platelets are the leading carrier of TGF‐β1 in blood and approximately 40% of the TGF‐β1 present in plasma is platelet‐derived [46], which is why plasma rather than serum is recommended for its measurement [47]. We minimized this contribution by preparing plasma within 30 min of venipuncture using two‐step centrifugation, but a residual platelet‐derived component cannot be excluded. Trophoblast‐derived cells are a further potential source, as progesterone increases their TGF‐β expression [23]. Second, circulating Tregs are an imperfect proxy for the maternal–fetal interface: fetus‐specific regulatory T cells migrate selectively from peripheral blood to the decidua in human pregnancy [48], and in a murine abortion‐prone model progesterone expanded the uterine Treg pool without altering peripheral Treg numbers [49]. Notably, progesterone drives the differentiation of naive cord blood T cells, but not of adult peripheral blood T cells, into suppressive Tregs [50], which is consistent with the absence of a change in maternal circulating Tregs in our cohort. Third, an increase in circulating TGF‐β1 does not by itself indicate increased TGF‐β signalling, since the effects of TGF‐β depend on the cellular context [9].

Notably, we observed an increase in TGF‐β1 concentrations following progesterone administration. This result is consistent with the findings of Yang et al. [51] in a mouse model, although similar observations have not yet been reported in humans. The immunological functions of progesterone are thought to be mediated by the progesterone‐induced blocking factor (PIBF), which is produced by PR‐positive lymphocytes during pregnancy as well as by several pregnancy‐associated tissues, including the human trophoblast, and by certain malignant tumours [52]. We are not yet able to precisely explain the origin of the increased TGF‐β1 concentrations observed after progesterone administration in women at risk of preterm birth. As mentioned above, no studies to date have specifically examined this relationship.

In addition, the study by Xie et al. may offer some insight. Their group evaluated hormone levels—including progesterone—in relation to the incidence of spontaneous abortion and confirmed a previously observed finding: women who experienced spontaneous abortion had significantly lower progesterone levels compared with those with normal pregnancies. Moreover, within the spontaneous abortion group, progesterone levels correlated positively with TGF‐β concentrations [53].

Another possible explanation was proposed by Vota et al. [32], who investigated whether progesterone can modulate trophoblast cell function, focusing on phagocytic capacity and the maintenance of immune homeostasis. Progesterone enhanced phagocytosis in trophoblast‐derived cells, quantified by the engulfment of latex beads or eryptotic erythrocytes. It also modulated the balance of anti‐inflammatory and pro‐inflammatory mediators, increasing TGF‐β expression, while no changes were observed in IL‐1, IL‐6, or nitrite production [32].

Other studies have also demonstrated that progesterone can enhance TGF‐β production, specifically in osteoblasts [54].

One possible interpretation of our findings is that vaginal progesterone may reinforce an immunoregulatory pathway that opposes, delays, or modulates components of the inflammatory program of parturition. This interpretation is biologically plausible in view of the evidence, summarized in the Introduction, that TGF‐β signalling is involved in several components of parturition [20, 21, 22, 23, 24, 25, 26]; however, the present study does not establish such a mechanism. TGF‐β1 was measured only in the maternal circulation, no untreated high‐risk group was included, and TGF‐β signalling in gestational tissues was not assessed. Moreover, TGF‐β should not be regarded simply as an anti‐inflammatory or tolerogenic cytokine, because its effects depend on the cellular context [20]. In CD4^+^ T cells, TGF‐β promotes the differentiation of iTregs, whereas in the presence of IL‐6 it drives the differentiation of Th17 cells, which can be pro‐inflammatory [10, 11]; in murine T cells, low concentrations of TGF‐β together with IL‐6 and IL‐21 favoured Th17 differentiation, whereas high concentrations favoured FoxP3^+^ Tregs [55]. In the human myometrium, the expression of TGF‐β receptors differs between the non‐pregnant state, pregnancy before labor, and spontaneous labor, suggesting that the myometrial TGF‐β signalling system is regulated across pregnancy and labor [20], and in human fetal membranes, TGF‐β reduced basal TNF‐α release but had little effect under lipopolysaccharide stimulation [24]. An increase in circulating TGF‐β1 therefore cannot be directly equated with an anti‐inflammatory effect at the maternal–fetal interface, and its biological consequences remain to be determined. This complexity makes the observed increase in TGF‐β1 during progesterone therapy all the more worth investigating in larger, adequately controlled studies.

We acknowledge that the small control cohort represents the main limitation of our study. The control group consisted of only eight women, which substantially limits the statistical power of the results. In the high‐risk cohort, blood samples were obtained from 21 women at the first collection, 17 at the second, and 7 at the third. In the control group, samples were available from 8, 6, and 5 women at the respective time points. This gradual reduction in sample size further limited the power of the results. An important limitation is the progressive reduction in the number of high‐risk participants contributing samples at later gestational time points. This attrition was not random because women who delivered before the scheduled sampling time could no longer provide a subsequent pregnancy sample. Consequently, the group represented at the third sampling time point is inherently enriched for pregnancies that continued to a later gestational age. Although mixed‐effects modeling allowed us to account for the unbalanced repeated‐measures design and a complete‐case sensitivity analysis was performed, these approaches cannot fully eliminate the possibility of selection bias related to pregnancy duration. Late‐pregnancy findings should therefore be interpreted in this context. In addition, because all high‐risk women received progesterone and no untreated group with a short cervix was available, the duration of progesterone therapy and advancing gestational age increased in parallel within this cohort; a progesterone‐associated effect therefore cannot be fully separated from a gestational‐age effect. Future studies should therefore aim to include larger and more balanced cohorts with complete longitudinal sampling to validate and extend these preliminary findings.

In summary, in this small exploratory cohort, vaginal progesterone treatment was accompanied by an increase in circulating TGF‐β1 that was not paralleled by an increase in circulating Tregs. Our data do not identify the cellular source of this increase, do not establish that it reflects increased TGF‐β signalling at the maternal–fetal interface, and do not allow any conclusion about the efficacy of progesterone in preventing preterm birth. They do, however, raise the possibility that progesterone influences an immunoregulatory pathway relevant to the transition between pregnancy maintenance and parturition. Testing this will require larger cohorts including an untreated comparison group, measurement of total and active TGF‐β1 together with markers of platelet activation, and, where feasible, assessment of TGF‐β signalling in gestational tissues.

## Author Contributions

Zdeněk Laštůvka and Viktor Černý: shared first authorship, conceptualization, methodology, investigation, funding acquisition, and original draft preparation. Anna Škvorová: formal analysis and original draft preparation. Olga Novotná and Helena Kopřivová: conceptualization, methodology, and writing – review and editing. Petra Petrásková: laboratory procedures. Michal Koucký and Jiří Hrdý: statistical analysis, funding acquisition, writing – review and supervision.

## Funding

The study was supported by MH CZ – DRO‐VFN00064165 and Charles University Research program Cooperatio IMMU207032.

## Ethics Statement

This research was approved by the Ethics Committee of the First Faculty of Medicine, Charles University, Prague, Czech Republic (No 481/19, S‐IV). The study was conducted in accordance with the Declaration of Helsinki principles.

## Consent

Informed consent was obtained from all participants.

## Conflicts of Interest

The authors declare no conflicts of interest.

## Data Availability

The data that support the findings of this study are available on request from the corresponding author. The data are not publicly available due to privacy or ethical restrictions.
